# (5-*n*-Heptyl-2-hydroxymethyl-1,3-dioxan-2-yl)methanol

**DOI:** 10.1107/S1600536810041413

**Published:** 2010-10-23

**Authors:** Xian-You Yuan, Min Zhang, Seik Weng Ng

**Affiliations:** a Department of Biology and Chemistry, Hunan University of Science and Engineering, Yongzhou Hunan 425100, People’s Republic of China; b Department of Chemistry, University of Malaya, 50603 Kuala Lumpur, Malaysia

## Abstract

In the title compound, C_13_H_26_O_4_, the dioxane rings adopts a chair conformation; the *n*-heptyl chain occupies an equatorial position. In the crystal, mol­ecules are connected by O—H⋯O hydrogen bonds into a zigzag chain running along the *a* axis, giving rise to a herringbone pattern. There are two independent mol­ecules in the asymmetric unit.

## Related literature

For a related structure, see: Luo *et al.* (2008 ▶).
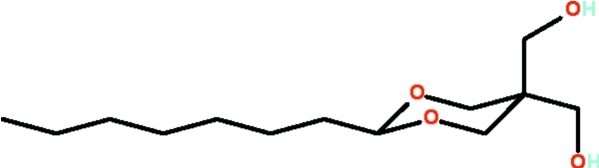


## Experimental

### 

#### Crystal data

C_13_H_26_O_4_
*M* *_r_* = 246.34
Monoclinic, 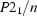

*a* = 5.8030 (4) Å
*b* = 54.017 (4) Å
*c* = 9.1018 (6) Å
β = 92.938 (1)°
*V* = 2849.3 (3) Å^3^
*Z* = 8
Mo *K*α radiation
μ = 0.08 mm^−1^
*T* = 163 K
0.40 × 0.35 × 0.15 mm

#### Data collection

Bruker SMART APEX diffractometer
14414 measured reflections
6156 independent reflections
3242 reflections with *I* > 2σ(*I*)
*R*_int_ = 0.077

#### Refinement

*R*[*F* ^2^ > 2σ(*F* ^2^)] = 0.077
*wR*(*F* ^2^) = 0.201
*S* = 1.05
6156 reflections
311 parameters
H-atom parameters constrained
Δρ_max_ = 0.32 e Å^−3^
Δρ_min_ = −0.32 e Å^−3^

### 

Data collection: *SMART* (Bruker, 2003 ▶); cell refinement: *SAINT* (Bruker, 2003 ▶); data reduction: *SAINT*; program(s) used to solve structure: *SHELXS97* (Sheldrick, 2008 ▶); program(s) used to refine structure: *SHELXL97* (Sheldrick, 2008 ▶); molecular graphics: *X-SEED* (Barbour, 2001 ▶); software used to prepare material for publication: *publCIF* (Westrip, 2010 ▶).

## Supplementary Material

Crystal structure: contains datablocks global, I. DOI: 10.1107/S1600536810041413/bt5378sup1.cif

Structure factors: contains datablocks I. DOI: 10.1107/S1600536810041413/bt5378Isup2.hkl

Additional supplementary materials:  crystallographic information; 3D view; checkCIF report

## Figures and Tables

**Table 1:** Hydrogen-bond geometry (Å, °)

*D*—H⋯*A*	*D*—H	H⋯*A*	*D*⋯*A*	*D*—H⋯*A*
O3—H3⋯O7	0.84	1.94	2.736 (3)	159
O4—H4⋯O3^i^	0.84	1.82	2.656 (3)	171
O7—H7⋯O8^ii^	0.84	1.82	2.646 (3)	167
O8—H8⋯O4	0.84	1.92	2.701 (3)	155

Symmetry codes: (i) 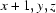; (ii) 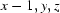.

**Fig. 1.**
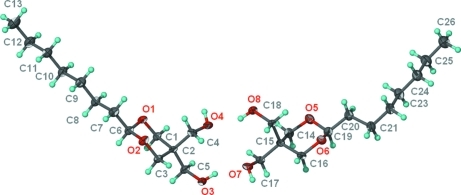
Anisotropic displacement ellipsoid plot (Barbour, 2001) of C13H26O4 at the 70% probability level; hydrogen atoms are shown as spheres of arbitrary radius.

**Fig. 2.**
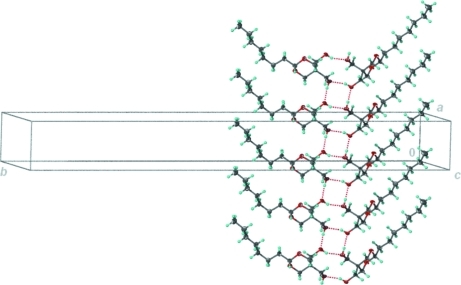
Partial packing diagram of the title compound showing the hydrogen-bonds as dashed lines.

## supplementary crystallographic information

### Comment

A previous study reported the crystal structure of 5,5-bis(hydroxylmethyl)-2-phenylmethyl-1,3-dioxane, which was synthesized by the condensation of 2,2-bis(hydroxymethyl)-1,3-propanediol and an aromatic aldehyde (benzaldehyde) (Luo *et al.*, 2008). A variation of the synthesis with an aliphatic aldehyde under similar reaction conditions yielded a 1,3-dixoxane having the hydroxyl groups connected to another atom of the chair-shaped ring. In the molecule of the C_13_H_26_O_4_ (Scheme I, Fig. 1), the *n*-heptyl chain  occupies an equatorial position. The   molecules are connected by O–H···O hydrogen-bonds to a zigzag chain running along the *a*-axis of the monoclinic unit cell giving rise to a herring-bone pattern. (Fig. 2).

### Experimental

2,2-Bis(hydroxymethyl)-1,3-propanediol (13.0 g, 96 mmol) and *N*,*N*-dimethylformamide (100 ml) were heated until the 2,2-bis(hydroxymethyl)-1,3-propanediol dissolved completely. *n*-Octanal (11.4 g, 89 mmol) and *p*-toluenesulfonic acid monohydrate (1 g, 5 mmol) were added. The solution was heated 363–373 K 5 h. The solution was cooled and ethyl acetate (100 ml) was added to dissolve the residue after DMF was removed by evaporation. The solution was washed successively with water and 5% sodium bicarbonate (50 ml); the solution was dried over sodium sulfate. The solvent was evaporated to give a solid that was recrystallized from ethyl acetate to yield 16.0 g (65%) of colorless crystals.

### Refinement

Carbon-bound H-atoms were placed in calculated positions (C—H 0.95–0.99 Å) and were included in the refinement in the riding model approximation, with *U*_iso_(H) set to 1.2–1.5*U*_eq_(C).

The hydroxy H-atoms were similarly placed (O–H 0.84%A) with *U*_iso_(H) set to  1.5*U*_eq_(O).

One of the axes of the unit cell is extremely long [54.017 (4) Å]; however, the crystal was sufficiently strongly diffracting at low temperature so that the refinement is stable, although it converged at a somewhat high *R* value.

### Crystal data

**Table:**

C_13_H_26_O_4_	*F*(000) = 1088
*M**_r_* = 246.34	*D*_x_ = 1.149 Mg m^−^^3^
Monoclinic, *P*2_1_/*n*	Mo *K*α radiation, λ = 0.71073 Å
Hall symbol: -P 2yn	Cell parameters from 2633 reflections
*a* = 5.8030 (4) Å	θ = 2.4–26.3°
*b* = 54.017 (4) Å	µ = 0.08 mm^−^^1^
*c* = 9.1018 (6) Å	*T* = 163 K
β = 92.938 (1)°	Prism, colorless
*V* = 2849.3 (3) Å^3^	0.40 × 0.35 × 0.15 mm
*Z* = 8	

### Data collection

**Table:**

Bruker SMART APEX diffractometer	3242 reflections with *I* > 2σ(*I*)
Radiation source: fine-focus sealed tube	*R*_int_ = 0.077
graphite	θ_max_ = 27.1°, θ_min_ = 2.3°
ω scans	*h* = −6→7
14414 measured reflections	*k* = −58→69
6156 independent reflections	*l* = −10→11

### Refinement

**Table:**

Refinement on *F*^2^	Primary atom site location: structure-invariant direct methods
Least-squares matrix: full	Secondary atom site location: difference Fourier map
*R*[*F*^2^ > 2σ(*F*^2^)] = 0.077	Hydrogen site location: inferred from neighbouring sites
*wR*(*F*^2^) = 0.201	H-atom parameters constrained
*S* = 1.05	*w* = 1/[σ^2^(*F*_o_^2^) + (0.0842*P*)^2^] where *P* = (*F*_o_^2^ + 2*F*_c_^2^)/3
6156 reflections	(Δ/σ)_max_ = 0.001
311 parameters	Δρ_max_ = 0.32 e Å^−^^3^
0 restraints	Δρ_min_ = −0.32 e Å^−^^3^

### Fractional atomic coordinates and isotropic or equivalent isotropic displacement parameters (Å^2^)

**Table:**

	*x*	*y*	*z*	*U*_iso_*/*U*_eq_	
O1	0.2272 (3)	0.13631 (4)	0.3678 (2)	0.0193 (5)	
O2	−0.0529 (4)	0.13323 (4)	0.1752 (2)	0.0230 (5)	
O3	−0.4134 (3)	0.19510 (4)	0.3742 (3)	0.0243 (6)	
H3	−0.3489	0.2088	0.3615	0.036*	
O4	0.1374 (4)	0.20487 (4)	0.3614 (3)	0.0281 (6)	
H4	0.2795	0.2022	0.3747	0.042*	
O5	0.1796 (4)	0.31378 (4)	0.4361 (2)	0.0247 (5)	
O6	−0.1028 (3)	0.32156 (4)	0.2493 (2)	0.0202 (5)	
O7	−0.3125 (4)	0.24297 (4)	0.3030 (3)	0.0254 (6)	
H7	−0.4518	0.2468	0.2868	0.038*	
O8	0.2389 (4)	0.25210 (4)	0.2915 (3)	0.0261 (6)	
H8	0.1682	0.2387	0.3026	0.039*	
C1	0.0843 (5)	0.15190 (5)	0.4533 (3)	0.0167 (7)	
H1A	0.1831	0.1635	0.5124	0.020*	
H1B	−0.0009	0.1415	0.5220	0.020*	
C2	−0.0868 (5)	0.16654 (5)	0.3557 (3)	0.0157 (7)	
C3	−0.2132 (5)	0.14847 (5)	0.2508 (4)	0.0212 (7)	
H3A	−0.3156	0.1378	0.3070	0.025*	
H3B	−0.3107	0.1579	0.1778	0.025*	
C4	0.0368 (5)	0.18620 (6)	0.2664 (4)	0.0224 (7)	
H4A	0.1590	0.1782	0.2110	0.027*	
H4B	−0.0750	0.1940	0.1946	0.027*	
C5	−0.2576 (5)	0.17895 (6)	0.4544 (4)	0.0208 (7)	
H5A	−0.3471	0.1660	0.5035	0.025*	
H5B	−0.1711	0.1885	0.5318	0.025*	
C6	0.0909 (5)	0.12001 (5)	0.2773 (3)	0.0184 (7)	
H6A	−0.0054	0.1094	0.3399	0.022*	
C7	0.2514 (6)	0.10397 (6)	0.1932 (4)	0.0243 (8)	
H7A	0.3531	0.1147	0.1369	0.029*	
H7B	0.1589	0.0937	0.1218	0.029*	
C8	0.4005 (5)	0.08698 (6)	0.2924 (4)	0.0231 (8)	
H8A	0.4899	0.0972	0.3655	0.028*	
H8B	0.2988	0.0759	0.3469	0.028*	
C9	0.5668 (6)	0.07125 (6)	0.2086 (4)	0.0270 (8)	
H9A	0.6675	0.0823	0.1534	0.032*	
H9B	0.4774	0.0609	0.1363	0.032*	
C10	0.7177 (6)	0.05444 (6)	0.3086 (4)	0.0278 (8)	
H10A	0.8078	0.0648	0.3803	0.033*	
H10B	0.6166	0.0436	0.3647	0.033*	
C11	0.8834 (6)	0.03837 (6)	0.2267 (4)	0.0312 (9)	
H11A	0.7934	0.0278	0.1561	0.037*	
H11B	0.9834	0.0492	0.1695	0.037*	
C12	1.0346 (6)	0.02205 (7)	0.3272 (5)	0.0397 (10)	
H12A	0.9345	0.0113	0.3851	0.048*	
H12B	1.1254	0.0327	0.3973	0.048*	
C13	1.1999 (7)	0.00570 (7)	0.2452 (5)	0.0547 (13)	
H13A	1.2931	−0.0042	0.3161	0.082*	
H13B	1.3015	0.0161	0.1888	0.082*	
H13C	1.1113	−0.0053	0.1781	0.082*	
C14	0.0664 (6)	0.29187 (6)	0.4811 (4)	0.0220 (7)	
H14A	−0.0366	0.2960	0.5610	0.026*	
H14B	0.1830	0.2799	0.5203	0.026*	
C15	−0.0747 (5)	0.28004 (5)	0.3539 (3)	0.0169 (7)	
C16	−0.2343 (5)	0.30020 (5)	0.2872 (4)	0.0211 (7)	
H16A	−0.3186	0.2937	0.1981	0.025*	
H16B	−0.3491	0.3049	0.3589	0.025*	
C17	−0.2122 (5)	0.25857 (5)	0.4161 (3)	0.0197 (7)	
H17A	−0.1088	0.2486	0.4824	0.024*	
H17B	−0.3363	0.2653	0.4752	0.024*	
C18	0.0813 (5)	0.27046 (5)	0.2361 (4)	0.0198 (7)	
H18A	−0.0156	0.2634	0.1538	0.024*	
H18B	0.1691	0.2845	0.1969	0.024*	
C19	0.0221 (5)	0.33143 (5)	0.3746 (4)	0.0208 (7)	
H19A	−0.0880	0.3365	0.4500	0.025*	
C20	0.1590 (6)	0.35353 (5)	0.3288 (4)	0.0241 (8)	
H20A	0.2648	0.3482	0.2529	0.029*	
H20B	0.2550	0.3594	0.4149	0.029*	
C21	0.0146 (6)	0.37513 (6)	0.2682 (4)	0.0258 (8)	
H21A	−0.0694	0.3826	0.3492	0.031*	
H21B	−0.1016	0.3688	0.1942	0.031*	
C22	0.1565 (6)	0.39521 (6)	0.1974 (4)	0.0243 (8)	
H22A	0.2339	0.3878	0.1134	0.029*	
H22B	0.0499	0.4082	0.1576	0.029*	
C23	0.3386 (6)	0.40747 (6)	0.2992 (4)	0.0276 (8)	
H23A	0.4417	0.3945	0.3427	0.033*	
H23B	0.2613	0.4156	0.3806	0.033*	
C24	0.4845 (6)	0.42659 (6)	0.2228 (4)	0.0292 (8)	
H24A	0.5621	0.4184	0.1414	0.035*	
H24B	0.3816	0.4396	0.1793	0.035*	
C25	0.6657 (6)	0.43875 (6)	0.3251 (4)	0.0333 (9)	
H25A	0.7675	0.4257	0.3690	0.040*	
H25B	0.5875	0.4469	0.4061	0.040*	
C26	0.8145 (7)	0.45783 (7)	0.2509 (5)	0.0425 (10)	
H26A	0.9262	0.4649	0.3237	0.064*	
H26B	0.7160	0.4711	0.2089	0.064*	
H26C	0.8969	0.4498	0.1724	0.064*	

### Atomic displacement parameters (Å^2^)

**Table:**

	*U*^11^	*U*^22^	*U*^33^	*U*^12^	*U*^13^	*U*^23^
O1	0.0133 (11)	0.0229 (11)	0.0217 (12)	0.0027 (9)	0.0007 (9)	−0.0051 (10)
O2	0.0214 (12)	0.0264 (12)	0.0208 (13)	0.0071 (10)	−0.0022 (9)	−0.0041 (10)
O3	0.0139 (11)	0.0199 (12)	0.0389 (15)	0.0018 (9)	0.0005 (10)	0.0019 (11)
O4	0.0125 (12)	0.0232 (12)	0.0488 (16)	−0.0011 (10)	0.0019 (11)	−0.0060 (11)
O5	0.0221 (12)	0.0233 (12)	0.0279 (14)	−0.0045 (10)	−0.0058 (10)	0.0055 (10)
O6	0.0183 (11)	0.0230 (11)	0.0190 (12)	−0.0029 (9)	−0.0038 (9)	−0.0013 (10)
O7	0.0126 (11)	0.0272 (12)	0.0363 (15)	−0.0046 (10)	0.0006 (10)	−0.0030 (11)
O8	0.0164 (12)	0.0223 (12)	0.0397 (15)	0.0025 (9)	0.0032 (10)	0.0043 (11)
C1	0.0169 (16)	0.0177 (16)	0.0155 (17)	0.0027 (13)	0.0005 (12)	−0.0030 (13)
C2	0.0099 (15)	0.0187 (16)	0.0185 (17)	0.0020 (12)	0.0000 (12)	−0.0003 (13)
C3	0.0155 (16)	0.0214 (16)	0.0268 (19)	0.0025 (13)	0.0017 (13)	−0.0042 (14)
C4	0.0150 (16)	0.0236 (17)	0.029 (2)	−0.0029 (14)	0.0016 (14)	−0.0007 (15)
C5	0.0166 (17)	0.0214 (16)	0.0245 (19)	0.0038 (13)	0.0018 (13)	0.0008 (14)
C6	0.0182 (17)	0.0201 (16)	0.0167 (17)	0.0025 (13)	−0.0005 (13)	−0.0025 (14)
C7	0.0239 (18)	0.0271 (18)	0.0220 (19)	0.0021 (14)	0.0018 (14)	−0.0042 (15)
C8	0.0229 (18)	0.0214 (17)	0.025 (2)	0.0039 (14)	0.0027 (14)	−0.0019 (14)
C9	0.0276 (19)	0.0283 (18)	0.026 (2)	0.0039 (15)	0.0046 (15)	−0.0028 (15)
C10	0.0253 (19)	0.0220 (17)	0.036 (2)	0.0049 (15)	0.0012 (16)	−0.0001 (16)
C11	0.0252 (19)	0.0247 (18)	0.044 (2)	0.0045 (15)	0.0056 (17)	−0.0003 (17)
C12	0.028 (2)	0.030 (2)	0.061 (3)	0.0070 (16)	0.0023 (19)	0.0008 (19)
C13	0.036 (2)	0.042 (2)	0.087 (4)	0.015 (2)	0.015 (2)	0.003 (2)
C14	0.0246 (18)	0.0225 (17)	0.0186 (18)	−0.0003 (14)	−0.0010 (14)	0.0023 (14)
C15	0.0129 (15)	0.0190 (16)	0.0186 (17)	−0.0002 (13)	−0.0007 (12)	0.0028 (13)
C16	0.0145 (16)	0.0221 (17)	0.0268 (19)	−0.0019 (13)	0.0022 (13)	0.0014 (14)
C17	0.0157 (16)	0.0213 (16)	0.0225 (19)	−0.0008 (13)	0.0051 (13)	0.0022 (14)
C18	0.0130 (16)	0.0189 (16)	0.0276 (19)	0.0016 (13)	0.0030 (13)	0.0016 (14)
C19	0.0203 (17)	0.0203 (17)	0.0214 (18)	−0.0009 (13)	−0.0036 (13)	−0.0030 (14)
C20	0.0266 (18)	0.0213 (17)	0.0244 (19)	0.0001 (14)	−0.0003 (14)	0.0026 (14)
C21	0.0263 (19)	0.0203 (17)	0.031 (2)	−0.0016 (14)	0.0005 (15)	0.0009 (15)
C22	0.0296 (19)	0.0197 (17)	0.0236 (19)	−0.0011 (14)	0.0030 (15)	0.0017 (14)
C23	0.033 (2)	0.0254 (18)	0.025 (2)	−0.0035 (15)	0.0024 (15)	0.0036 (15)
C24	0.032 (2)	0.0276 (19)	0.028 (2)	0.0004 (16)	0.0066 (16)	0.0041 (16)
C25	0.032 (2)	0.0304 (19)	0.037 (2)	−0.0052 (16)	0.0015 (17)	0.0010 (17)
C26	0.040 (2)	0.034 (2)	0.054 (3)	−0.0042 (18)	0.009 (2)	0.0079 (19)

### Geometric parameters (Å, °)

**Table:**

O1—C6	1.418 (4)	C11—H11A	0.9900
O1—C1	1.438 (3)	C11—H11B	0.9900
O2—C6	1.411 (4)	C12—C13	1.527 (5)
O2—C3	1.443 (3)	C12—H12A	0.9900
O3—C5	1.430 (4)	C12—H12B	0.9900
O3—H3	0.8400	C13—H13A	0.9800
O4—C4	1.433 (4)	C13—H13B	0.9800
O4—H4	0.8400	C13—H13C	0.9800
O5—C19	1.417 (4)	C14—C15	1.524 (4)
O5—C14	1.423 (3)	C14—H14A	0.9900
O6—C19	1.423 (4)	C14—H14B	0.9900
O6—C16	1.435 (3)	C15—C18	1.529 (4)
O7—C17	1.431 (4)	C15—C17	1.533 (4)
O7—H7	0.8400	C15—C16	1.534 (4)
O8—C18	1.424 (4)	C16—H16A	0.9900
O8—H8	0.8400	C16—H16B	0.9900
C1—C2	1.520 (4)	C17—H17A	0.9900
C1—H1A	0.9900	C17—H17B	0.9900
C1—H1B	0.9900	C18—H18A	0.9900
C2—C3	1.526 (4)	C18—H18B	0.9900
C2—C5	1.527 (4)	C19—C20	1.505 (4)
C2—C4	1.537 (4)	C19—H19A	1.0000
C3—H3A	0.9900	C20—C21	1.523 (4)
C3—H3B	0.9900	C20—H20A	0.9900
C4—H4A	0.9900	C20—H20B	0.9900
C4—H4B	0.9900	C21—C22	1.525 (4)
C5—H5A	0.9900	C21—H21A	0.9900
C5—H5B	0.9900	C21—H21B	0.9900
C6—C7	1.509 (4)	C22—C23	1.521 (4)
C6—H6A	1.0000	C22—H22A	0.9900
C7—C8	1.525 (4)	C22—H22B	0.9900
C7—H7A	0.9900	C23—C24	1.526 (4)
C7—H7B	0.9900	C23—H23A	0.9900
C8—C9	1.520 (4)	C23—H23B	0.9900
C8—H8A	0.9900	C24—C25	1.518 (5)
C8—H8B	0.9900	C24—H24A	0.9900
C9—C10	1.529 (4)	C24—H24B	0.9900
C9—H9A	0.9900	C25—C26	1.524 (5)
C9—H9B	0.9900	C25—H25A	0.9900
C10—C11	1.519 (4)	C25—H25B	0.9900
C10—H10A	0.9900	C26—H26A	0.9800
C10—H10B	0.9900	C26—H26B	0.9800
C11—C12	1.517 (5)	C26—H26C	0.9800
			
C6—O1—C1	110.9 (2)	H13A—C13—H13B	109.5
C6—O2—C3	110.4 (2)	C12—C13—H13C	109.5
C5—O3—H3	109.5	H13A—C13—H13C	109.5
C4—O4—H4	109.5	H13B—C13—H13C	109.5
C19—O5—C14	112.0 (2)	O5—C14—C15	111.6 (2)
C19—O6—C16	111.3 (2)	O5—C14—H14A	109.3
C17—O7—H7	109.5	C15—C14—H14A	109.3
C18—O8—H8	109.5	O5—C14—H14B	109.3
O1—C1—C2	111.5 (2)	C15—C14—H14B	109.3
O1—C1—H1A	109.3	H14A—C14—H14B	108.0
C2—C1—H1A	109.3	C14—C15—C18	111.1 (2)
O1—C1—H1B	109.3	C14—C15—C17	107.8 (2)
C2—C1—H1B	109.3	C18—C15—C17	110.0 (2)
H1A—C1—H1B	108.0	C14—C15—C16	107.1 (2)
C1—C2—C3	108.1 (2)	C18—C15—C16	109.2 (3)
C1—C2—C5	108.0 (2)	C17—C15—C16	111.6 (2)
C3—C2—C5	110.1 (2)	O6—C16—C15	110.4 (2)
C1—C2—C4	111.1 (2)	O6—C16—H16A	109.6
C3—C2—C4	109.5 (2)	C15—C16—H16A	109.6
C5—C2—C4	110.0 (2)	O6—C16—H16B	109.6
O2—C3—C2	111.2 (2)	C15—C16—H16B	109.6
O2—C3—H3A	109.4	H16A—C16—H16B	108.1
C2—C3—H3A	109.4	O7—C17—C15	112.3 (2)
O2—C3—H3B	109.4	O7—C17—H17A	109.1
C2—C3—H3B	109.4	C15—C17—H17A	109.1
H3A—C3—H3B	108.0	O7—C17—H17B	109.1
O4—C4—C2	110.8 (3)	C15—C17—H17B	109.1
O4—C4—H4A	109.5	H17A—C17—H17B	107.9
C2—C4—H4A	109.5	O8—C18—C15	112.3 (3)
O4—C4—H4B	109.5	O8—C18—H18A	109.1
C2—C4—H4B	109.5	C15—C18—H18A	109.1
H4A—C4—H4B	108.1	O8—C18—H18B	109.1
O3—C5—C2	112.3 (3)	C15—C18—H18B	109.1
O3—C5—H5A	109.1	H18A—C18—H18B	107.9
C2—C5—H5A	109.1	O5—C19—O6	110.9 (2)
O3—C5—H5B	109.1	O5—C19—C20	107.7 (2)
C2—C5—H5B	109.1	O6—C19—C20	109.3 (3)
H5A—C5—H5B	107.9	O5—C19—H19A	109.6
O2—C6—O1	111.2 (2)	O6—C19—H19A	109.6
O2—C6—C7	108.4 (2)	C20—C19—H19A	109.6
O1—C6—C7	108.1 (2)	C19—C20—C21	114.8 (3)
O2—C6—H6A	109.7	C19—C20—H20A	108.6
O1—C6—H6A	109.7	C21—C20—H20A	108.6
C7—C6—H6A	109.7	C19—C20—H20B	108.6
C6—C7—C8	113.0 (3)	C21—C20—H20B	108.6
C6—C7—H7A	109.0	H20A—C20—H20B	107.5
C8—C7—H7A	109.0	C20—C21—C22	113.5 (3)
C6—C7—H7B	109.0	C20—C21—H21A	108.9
C8—C7—H7B	109.0	C22—C21—H21A	108.9
H7A—C7—H7B	107.8	C20—C21—H21B	108.9
C9—C8—C7	113.3 (3)	C22—C21—H21B	108.9
C9—C8—H8A	108.9	H21A—C21—H21B	107.7
C7—C8—H8A	108.9	C23—C22—C21	115.2 (3)
C9—C8—H8B	108.9	C23—C22—H22A	108.5
C7—C8—H8B	108.9	C21—C22—H22A	108.5
H8A—C8—H8B	107.7	C23—C22—H22B	108.5
C8—C9—C10	113.1 (3)	C21—C22—H22B	108.5
C8—C9—H9A	109.0	H22A—C22—H22B	107.5
C10—C9—H9B	109.0	C22—C23—C24	113.7 (3)
C8—C9—H9A	109.0	C22—C23—H23A	108.8
C10—C9—H9A	109.0	C24—C23—H23A	108.8
H9A—C9—H9B	107.8	C22—C23—H23B	108.8
C11—C10—C9	113.9 (3)	C24—C23—H23B	108.8
C11—C10—H10A	108.8	H23A—C23—H23B	107.7
C9—C10—H10A	108.8	C25—C24—C23	113.4 (3)
C11—C10—H10B	108.8	C25—C24—H24A	108.9
C9—C10—H10B	108.8	C23—C24—H24A	108.9
H10A—C10—H10B	107.7	C25—C24—H24B	108.9
C12—C11—C10	113.4 (3)	C23—C24—H24B	108.9
C12—C11—H11A	108.9	H24A—C24—H24B	107.7
C10—C11—H11A	108.9	C24—C25—C26	114.3 (3)
C12—C11—H11B	108.9	C24—C25—H25A	108.7
C10—C11—H11B	108.9	C26—C25—H25A	108.7
H11A—C11—H11B	107.7	C24—C25—H25B	108.7
C11—C12—C13	113.6 (3)	C26—C25—H25B	108.7
C11—C12—H12A	108.9	H25A—C25—H25B	107.6
C13—C12—H12A	108.9	C25—C26—H26A	109.5
C11—C12—H12B	108.9	C25—C26—H26B	109.5
C13—C12—H12B	108.9	H26A—C26—H26B	109.5
H12A—C12—H12B	107.7	C25—C26—H26C	109.5
C12—C13—H13A	109.5	H26A—C26—H26C	109.5
C12—C13—H13B	109.5	H26B—C26—H26C	109.5
			
C6—O1—C1—C2	−56.8 (3)	C19—O5—C14—C15	57.2 (3)
O1—C1—C2—C3	51.2 (3)	O5—C14—C15—C18	66.1 (3)
O1—C1—C2—C5	170.2 (2)	O5—C14—C15—C17	−173.3 (2)
O1—C1—C2—C4	−69.0 (3)	O5—C14—C15—C16	−53.1 (3)
C6—O2—C3—C2	57.9 (3)	C19—O6—C16—C15	−59.0 (3)
C1—C2—C3—O2	−51.7 (3)	C14—C15—C16—O6	53.9 (3)
C5—C2—C3—O2	−169.5 (2)	C18—C15—C16—O6	−66.5 (3)
C4—C2—C3—O2	69.5 (3)	C17—C15—C16—O6	171.7 (2)
C1—C2—C4—O4	−66.5 (3)	C14—C15—C17—O7	−167.6 (2)
C3—C2—C4—O4	174.1 (2)	C18—C15—C17—O7	−46.3 (3)
C5—C2—C4—O4	53.0 (3)	C16—C15—C17—O7	75.1 (3)
C1—C2—C5—O3	174.5 (2)	C14—C15—C18—O8	60.5 (3)
C3—C2—C5—O3	−67.6 (3)	C17—C15—C18—O8	−58.8 (3)
C4—C2—C5—O3	53.1 (3)	C16—C15—C18—O8	178.4 (2)
C3—O2—C6—O1	−62.5 (3)	C14—O5—C19—O6	−59.6 (3)
C3—O2—C6—C7	178.8 (2)	C14—O5—C19—C20	−179.1 (2)
C1—O1—C6—O2	62.1 (3)	C16—O6—C19—O5	60.7 (3)
C1—O1—C6—C7	−179.0 (2)	C16—O6—C19—C20	179.3 (2)
O2—C6—C7—C8	−174.7 (2)	O5—C19—C20—C21	−176.5 (3)
O1—C6—C7—C8	64.6 (3)	O6—C19—C20—C21	62.9 (3)
C6—C7—C8—C9	−178.5 (3)	C19—C20—C21—C22	−169.5 (3)
C7—C8—C9—C10	179.4 (3)	C20—C21—C22—C23	−60.4 (4)
C8—C9—C10—C11	179.4 (3)	C21—C22—C23—C24	177.3 (3)
C9—C10—C11—C12	179.2 (3)	C22—C23—C24—C25	180.0 (3)
C10—C11—C12—C13	179.5 (3)	C23—C24—C25—C26	179.7 (3)

### Hydrogen-bond geometry (Å, °)

**Table:**

*D*—H···*A*	*D*—H	H···*A*	*D*···*A*	*D*—H···*A*
O3—H3···O7	0.84	1.94	2.736 (3)	159
O4—H4···O3^i^	0.84	1.82	2.656 (3)	171
O7—H7···O8^ii^	0.84	1.82	2.646 (3)	167
O8—H8···O4	0.84	1.92	2.701 (3)	155

Symmetry codes: (i) *x*+1, *y*, *z*; (ii) *x*−1, *y*, *z*.

## References

[bb1] Barbour, L. J. (2001). *J. Supramol. Chem.* **1**, 189–191.

[bb2] Bruker (2003). *SAINT* and *SMART* Bruker AXS Inc., Madison, Wisconsin, USA.

[bb3] Luo, Y.-M., Liu, X.-M., Yuan, X.-Y., Zhang, M. & Ng, S. W. (2008). *Acta Cryst.* E**64**, o1536. 10.1107/S1600536808022046 PMC2962161 21203241

[bb4] Sheldrick, G. M. (2008). *Acta Cryst.* A**64**, 112–122. 10.1107/S0108767307043930 18156677

[bb5] Westrip, S. P. (2010). *J. Appl. Cryst.* **43**, 920–925.

